# Supplementary material for: An in-depth examination of the fuzzy fractional cancer tumor model and its numerical solution by implicit finite difference method
Source: PLoS One. 2024 Dec 20;19(12):e0303891. doi: 10.1371/journal.pone.0303891 (PMC11661611; doi:10.1371/journal.pone.0303891)
Supplement: Attachment — Submitted filename: Response to Reviewers 2.docx [file pone.0303891.s004.docx]

Firstly, the authors would like to express their gratitude to the reviewer for his invaluable feedback and comments. In response, we have added a two paragraphs at the end of the introduction (Section 1) to clarify the differences between our work and the other articles in particular the article (DOI: 10.3390/ijerph20043766), and to explain what specific gap in this field that the paper filled.

**For more details, please read the following:**

In the context of time fractional cancer tumor models, there's a need to introduce fuzzy quantities instead of crisp quantities to accommodate the inherent uncertainty and imprecision in this model, giving rise to a formulation known as fuzzy time fractional cancer tumor models. Therefore, we discussed this issue in the article (DOI: 10.3390/ijerph20043766) where we addressed, developed and applied an explicit method to solve the fuzzy fractional cancer tumor model. The authors discussed the Stability Analysis for the explicit method in (section 4), and they found that the system is conditionally stable, indicating its inability to include all scenarios. This becomes obvious in the article, wherein the authors restrict their discussion to a few specific cases of the presented model and in all of them the study cases α≥ 0.5 while they do not discuss the cases when α is less than 0.5 or tends towards zero. Contrastingly, the authors of this paper resolved this issue by employing an implicit method to solve the fuzzy fractional cancer tumor model, as delineated in Section 4 (Stability Analysis), the authors showed that the system is unconditionally stable indicating its ability to include all scenarios and capable of encompassing all potential cases and this can be seen in section 5 where the authors studied all cases.

The two articles take part with each other in Section 2, which is required for discussion in all research articles that discuss cancer tumor models in a fuzzy environment. However, the other sections of our article differ from the one mentioned (10.3390/ijerph20043766).
